# Evaluation of Electroencephalography, Behaviour and Eye Temperature in Response to Surgical Castration in Sheep

**DOI:** 10.3390/ani11030637

**Published:** 2021-02-27

**Authors:** Charissa Harris, Peter John White, Evelyn Hall, Dominique Van der Saag, Sabrina Lomax

**Affiliations:** 1 School of Life and Environmental Sciences, Faculty of Science, The University of Sydney, Sydney 2006, Australia; char2922@uni.sydney.edu.au; 2 Sydney School of Veterinary Science, Faculty of Science, The University of Sydney, Sydney 2006, Australia; p.white@sydney.edu.au (P.J.W.); evelyn.hall@sydney.edu.au (E.H.); dominique.van.der.saag@sydney.edu.au (D.V.d.S.)

**Keywords:** electroencephalography, castration, anaesthesia, sheep, pain

## Abstract

**Simple Summary:**

Australian sheep undergo several surgical procedures as per industry standard lamb marking, including surgical castration. Electroencephalography (EEG) has been used successfully to assess pain in sheep previously. In this study, the EEG was measured over time to assess castration pain and analgesic efficacy in conscious lambs in combination with behaviour observations and eye temperature. A combination of lignocaine and meloxicam was used as the gold-standard approach to pain relief (CML), compared to castrated but untreated (C) and sham castrated (SC) lambs. Changes in EEG and behaviour were observed in all treatment groups. Lambs in CML and C groups showed more abnormal behaviours and immediate increases in eye temperature, indicative of pain. However, little differences between these groups suggest that analgesia was not successful in removing perceived pain of the procedure in this study. Changes in EEG for SC groups suggests the effect of handling and restraint, in addition to the pain of castration, impacted all results, not singularly the pain of castration.

**Abstract:**

Castration has been demonstrated to cause pain in sheep. However, it is routinely performed for management purposes. Electroencephalography (EEG) has been used successfully to measure pain in lambs in response to castration and other husbandry procedures in livestock. The aim of this study was to evaluate the use of EEG as a measure of pain and analgesia in conscious lambs undergoing castration on farm over a 24 h period. EEG responses were compared to behavioural observations and changes in ocular temperature via infrared thermography. Twenty-four merino ram lambs (18.63 ± 2.06 kg) were used in this study. Lambs were randomly allocated to one of the following treatment groups: (1) castration with pre-surgical administration of meloxicam (0.5 mg/kg Metacam 20 mg/mL injected subcutaneously into the skin of the neck 15 min prior to recording) and lignocaine (applied via intra-testicular injection five minutes prior to castration, 2 mL lignocaine hydrochloride 20 mg/mL, Troy/Ilium) (CML, *n* = 8); (2) castration only (C, *n* = 8); (3) sham castration, handling only (SC, *n* = 8). EEG was recorded for 5 mins pre-procedure (prior to any intervention), and for 5 mins post-procedure at 0, 1, 4 and 24 h. Behavioural reactions to the procedure were scored, and behaviours were scan sampled at 5 min intervals at the above time points, by blinded observers. Eye temperature was measured for five-minute intervals at each time point. EEG decreased from baseline to 0 h for CML and C groups (*p* < 0.001), C group values returned similar to baseline at 24 h. Eye temperature increased post-castration at 0 h for C group, no initial change was seen for CML or SC groups. CML and C groups were more likely to have higher reaction scores and showed more abnormal behaviours (*p* = 0.017). CML and C groups had similar results, indicating minimal effect of analgesic intervention. Lambs in SC group showed significant EEG changes, suggesting that stress from handling also impacted EEG results.

## 1. Introduction

Castration of sheep is routinely performed to control unwanted breeding in extensively managed flocks as well as to improve handling. It is widely accepted to be a painful procedure. However, it is deemed necessary for animal management, justifying its continued use on farms throughout Australia. Australian animal welfare standards allow castration to be performed without pain relief on sheep of six months of age or less [1], despite evidence that lambs experience pain from 5 days of age [2]. Castration is commonly performed using a surgical knife or rubber ring. Both methods cause an increase in plasma cortisol concentrations and abnormal behaviours in lambs for up to four hours post-procedure [3]. As pain and distress caused by castration have been well documented in the literature, current research is focused on evaluating options for effective pain mitigation.

Pain and analgesia in castrated lambs have been examined using several physiological and behavioural parameters with varying success. Blood-based parameters such as plasma cortisol have been used to assess pain caused by castration [4,5]. An increase in cortisol was indicative of a pain response to castration in lambs and was able to rank castration procedures on a pain scale [6]. However, increases in cortisol have also been reported in control animals due to handling stress [6,7] and as a response to surgical wounding and resulting blood loss rather than pain [8]. Objective, non-invasive measures of pain offer insight into animal response while minimising confounding factors. Acute behavioural responses to castration in sheep can be greatly reduced with the use of analgesic intervention. Small et al. [9] noted a 7-fold reduction in abnormal behaviours with the use of a meloxicam formulated for oral-mucosal absorption. Similarly, application of a topical anaesthetic formulation reduced the pain-related behaviours exhibited by surgically castrated lambs [10]. Both eye temperature and heart rate have been used to assess pain responses in livestock in response to surgical procedures. Changes in eye temperature measured with infrared thermography have been shown to reflect pain after disbudding in dairy calves [11,12]. Individual measures of pain can give varying results depending on study design and compounding factors and so a combination of parameters is likely to offer the best and most reliable overview of an animal’s experience. Electroencephalography (EEG) has been used to assess pain responses in lambs undergoing castration [13,14,15]. Studies evaluating changes in EEG output after painful and stressful events in conscious sheep suggest that EEG can provide insight into a pain response through objective numeric measurements [16,17].

The objective of this study was to evaluate the use of EEG as a measure of pain and analgesia in conscious lambs undergoing castration on farm over a 24 h period. Previously published research has demonstrated that EEG was able to distinguish between lambs castrated with or without pain relief immediately post-surgery [13], and therefore the aim of this study was to replicate that in a field setting over a longer duration relevant to castration pain and analgesic efficacy. A multi-parametric approach to pain assessment was evaluated in comparison with the EEG output in this study.

## 2. Materials and Methods

This experiment was conducted at The University of Sydney’s commercial Merino property in the Southern Highlands of NSW, Australia. The experimental protocol was approved by the University of Sydney’s animal ethics committee (AEC approval number 2019/1624). This study was conducted across 3 days in November 2019.

### 2.1. Animal Handling and Management

Twenty-four merino ram lambs (18.63 ± 2.06 kg) were used for this study and housed under cover in a shearing shed in group pens (2 m × 2 m) with access to feed and water. Prior to the study lambs were maintained with their mothers on the farm before being mustered and drafted from the flock for the duration of this study. Prior to data collection, all lambs were weighed using standard bathroom scales and each lamb was painted with a number (1 to 24) on each side and rump using stock mark spray for video identification. Data were collected immediately prior to castration (pre), immediately after (0 h) and at 1, 4 and 24 h after. Lambs were returned to their mothers and the flock on the same day the 24 h time point data were collected.

### 2.2. Experimental Design

The experiment was conducted in two blocks over 3 days, so that on Day 1 twelve lambs were treated, with 4 h of observations recorded before lambs were returned to their pens. A final 24 h observation for this group was collected on Day 2. On Day 2, the remaining 12 lambs were treated, with their 24 h observations collected on Day 3. Lambs for each treatment group were evenly represented across treatment days and blocks to account for variations in environment. No lambs included in this study had previously experience EEG recording.

Lambs were allocated to one of three treatment groups using computer-generated random numbers (Excel^®^ version 16, © Microsoft corporation, International, 2018) with treatments blocked over two days and two time periods. Treatment groups were as follows: (1) castration with pre-surgical administration of meloxicam 15 min prior to recording (0.5 mg/kg Metacam 20 mg/mL injected subcutaneously into the skin of the neck) and lignocaine applied via intra-testicular injection five minutes prior to incision (2 mL lignocaine hydrochloride 20 mg/mL, Troy/Ilium) (CML, *n* = 8); (2) castration only, no treatment (C, *n* = 8); (3) sham castration, handling only (SC, *n* = 8). EEG was recorded for 5 mins pre-procedure (prior to any intervention), and for 5 mins post-procedure at 0, 1, 4 and 24 h.

### 2.3. Castration

Surgical knife castration was performed by a single, experienced technician. A standard sharpened lamb-marking knife was used for the procedure which was disinfected between lambs using 8 g/L chlorhexidine gluconate (Hibitane disinfectant, Coopers^®^ Animal Health Australia, Canberra, NSW, Australia) diluted in water (100 mL/L). Castration involved the following steps: (1) disinfection and excision of the distal third of the scrotum to expose the testes; (2) extraction of each individual testicle from the scrotum by traction, thereby exposing the spermatic cord; (3) removal of the testis using tension and twisting of the spermatic cord so that the tissue was torn to aid in haemostasis; (4) cleaning of the surgical knife with disinfectant between lambs. Sham control lambs were handled in a manner as close as possible to castrated lambs without any surgical intervention, so that handling was kept consistent. Once in the cradle, the scrotum of sham lambs was manipulated by applying tension to scrotum and palpation of the testes.

### 2.4. EEG and HR Recording

EEG recording was performed as described by Harris et al. [13] using Labchart and Video Capture (Lab Chart ADInstruments Ltd., Bella Vista, NSW, Australia) with continuous live recording for data collection. Data cleaning and analysis was performed offline after the completion of the experiment. Data epochs containing movement artefacts were excluded from analysis. Data points excluded from analysis were restricted to 1–3 s spurious noise artefacts caused directly by subject movement, confirmed by video footage, including head tossing or whole-body flinching. EEG set up was adapted from Mayhew and Washbourne, where a three-electrode montage of 12 mm monopolar needle electrodes (29 gauge) made from surgical steel (ADInstruments Ltd., Bella Vista, NSW, Australia) were used to record EEG as follows: the non-inverting electrode was placed in the midline over the frontal sinus, the inverting electrode over the right mastoid process, and the common electrode caudal to the occipital process. Data for each time point were examined in two-second epochs and Fast Fourier Transformation was used to derive a power spectrum from which statistical descriptors of the median frequency (F50), the spectral edge frequency (F95) and the total power under the curve (Ptot) were derived for further analysis.

Electrocardiograms were recorded using Labchart software (Lab Chart ADInstruments Ltd., Bella Vista, NSW, Australia) using a three-electrode montage with active electrode on the left fore flank, reference electrode on the inside of the right hind limb, with the ground electrode common to the EEG [13].

Baseline recordings of five minutes were taken prior to castration for both EEG and ECG, a further five minutes was recorded post-castration or sham handling, and for five minutes at each time point thereafter.

### 2.5. Reaction Score to Castration

Lambs were recorded in the cradle for the duration of the castration procedure using a handheld digital SLR camera (DSLR 500, Canon, Tokyo, Japan). Videos were viewed after the completion of this study to score the reaction of each lamb to the procedure. Two experienced observers, blinded to treatments, recorded their observations for each lamb by categorising reactions on a numerical scale, adapted from Mellema et al. and Lomax et al. [10,18], for overall reaction to the procedure, reaction to distal scrotal incision and for each testis removal, with testis 1 and 2 being the right and left testis, respectively (Table 1).

### 2.6. Infrared Thermography

A handheld infrared thermography camera (FLIRE50, FLIR Systems, Inc., International, Mulgrave, VIC, Australia) was used to record eye temperature of lambs continuously for 5 min periods during EEG data collection at each time point of baseline, 0, 1, 4, and 24 h. The infrared camera was positioned so that marker points within the camera view were aligned with the centre of the lambs’ cornea keeping a consistent distance from the animal of approximately 30 cm. Ambient temperature and humidity were monitored, and the values entered into the camera for calibration at each time point. Videos were analysed at a later date and maximum eye temperature was recorded at 20 s intervals.

### 2.7. Behaviour Observation Recordings

As soon as the EEG and IRT recordings were complete, lambs were moved to small holding pens (3 m × 3 m) in groups of six. Lambs were kept in these holding pens between data collection times for video recordings. Wide-angle video cameras (Sony Australia Ltd., North Sydney, NSW, Australia) were mounted on each pen for continuous recording of behaviour across 4 time points post-castration. Behaviours were analysed after completion of the experiment using the video recordings. Behaviours were recorded using a customised ethogram (Table 2) adapted from two studies conducted by Small et al. [9,19]. Scan sampling was conducted at five minute intervals for one hour periods post-castration at 0, 1, 4, 24 h [20]. Lambs were identified by the painted number and their behaviour was then recorded for that scan time as ventral lying (normal, abnormal or other), lateral lying, standing (normal, hunched or abnormal), walking (normal, abnormal or other) or eating/drinking. Two observers, blinded to treatments, conducted the behavioural observations using the established ethogram (Table 2) after conclusion of the experiment with observations recorded directly into excel. Observers received training prior to data recording to standardise their observations. An inter-observer reliability test was conducted by viewing six set videos for fifteen minutes showing examples of behaviours. Observers recorded a 94% concordance in behaviour identification for the test videos. Video footage where a handler was present in the pen was disregarded to avoid recording behaviour in response to human presence. In these cases, behaviour recording resumed after lambs had ceased reacting to the pen disturbance. Due to no or few occurrences of some behaviours, including ‘other walking’, ‘other ventral lying’ and ‘lateral lying’, ethogram categories were grouped into broader categories of ‘normal’ and ‘abnormal’ behaviours for walking, lying and standing for analysis. A further grouping was made that included all abnormal data for walking, lying and standing, and the same for normal, and classified ‘total normal’ and ‘total abnormal’.

### 2.8. Statistical Analysis

EEG data were exported from Labchart^®^ software (LabChart Pro, version 8, AD Instruments Ltd., Bella Vista, NSW, Australia) into Matlab^®^ (version R2017b, MathWorks, Inc. Natick, MA, USA) for further analysis as described by Harris et al. [13]. EEG and IRT data were analysed in Rstudio (Version 1.1.447—© 2009–2018 RStudio, Inc., Boston, MA, USA) with linear mixed models, using the ‘lme4′ package. EEG data Ptot and F95 data were normalised using a natural logarithmic scale where necessary. The fixed effects of the linear mixed model were Treatment and Time point, with random effects being Lamb and Day. The effect of Treatment on Castration reaction scores was analysed by ordinal logistic regression (clm) within the ‘ordinal’ package in Rstudio. Behaviour data were also analysed in Rstudio using generalised linear mixed model (glmer) with binomial proportions. Fixed effects were Treatment and Time, with a random term of Lamb included. Behaviours were examined individually for treatment by time interactions and grouped into normal and abnormal categories for statistical analysis. For all data analysis, *p* values of <0.05 were considered statistically significant.

## 3. Results

### 3.1. Electroencephalography

There were significant treatment x time interactions (*p* < 0.001) across all treatment groups for F50, F95 and Ptot measures. Back-transformed mean values and interactions are presented below (Table 3). There was an initial decrease from pre to 0 h across all EEG parameters for treatments CML and C. There was no difference between pre and 24 h values for F50, F95 and Ptot for lambs in treatment C. This trend also occurred in F95 for SC and Ptot for CML lambs. Lambs in the SC group did not display any changes from baseline to 0 h for F50.

### 3.2. Infrared Thermography of the Eye

**Figure 1:**
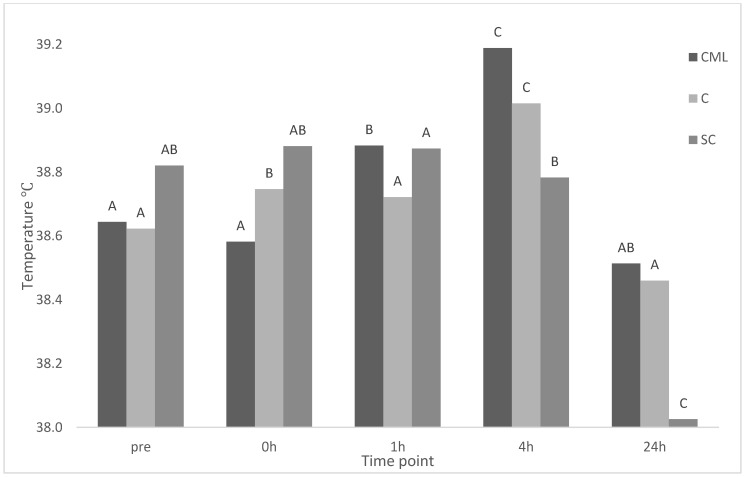
Maximum eye temperature of treatment groups castration + analgesia (CML), castration (C), and sham control (SC) (*n* = 8 for all treatments) across time points pre (baseline), 0, 1, 4, 24 h. Means between time points within a treatment group (column) with differing superscripts ^ABC^ are significantly different.

There was a significant treatment × time interaction for eye temperature (*p* < 0.001) (Figure 1). There was no effect of treatment on eye temperature at any time point. There was no change in eye temperature for CML lambs from baseline to immediately after castration. A significant increase occurred at 1 h and again at 4 h before decreasing at 24 h back to a temperature similar to baseline. There was a significant increase in eye temperature immediately post-castration at 0 h, and a further increase at 4 h, with temperatures similar to baseline at 1 and 24 h for C lambs. There was no difference from baseline to 1 h before significantly decreasing for further time points for SC lambs.

### 3.3. Reaction Scores to Castration

There was a significant effect of treatment on the behavioural response to scrotal incision (*p* < 0.001). The SC group was most likely to not respond (score 0) to scrotal manipulation compared to CML and C groups (Table 4). A significant effect of treatment was observed for both removal of testes 1 and 2 (*p* < 0.01 and *p* = 0.04, respectively). Regarding removal of the first testis (right), SC group were more likely to score 0, C group were more likely to receive the highest score of 3, and CML a score of 2 (Table 4). For overall reaction to the procedure (Table 4), no treatment effect was found (*p* = 0.1). However, the trend of SC group lambs scoring lower than C and CML groups was still apparent.

### 3.4. Behaviour Analysis

*p* value results from analysis for grouped categories for behaviours are presented below (Table 5, Table 6, Table 7 and Table 8). There was a significant effect of treatment on the proportion of time an abnormal behaviour was observed during sampling period (*p* = 0.017) (Table 5). Significant interactions of treatment and time were seen for Normal lying (*p* < 0.001) and Normal standing (*p* = 0.002) (Table 6). Lambs in the CML and C treatment groups displayed Total abnormal behaviour significantly more than SC (Table 8).

## 4. Discussion

This study examined the use of EEG in combination with behavioural and physiological parameters to attempt to measure the pain of surgical castration in lambs, with and without multi-modal analgesic intervention. Experimental factors including animal handling likely influenced the outcome of this study with no significant effects distinguishing between treatment groups, with the exception of select behaviours. Electroencephalography output changed significantly over time within treatment groups. This suggests that a stress response to handling, as well as procedural pain in castrated lambs, was detected. To the authors’ knowledge, this is the only study that has investigated a multi-parametric approach to examining castration in conscious sheep with the inclusion of EEG as a measure.

There were significant changes in EEG parameters across time points in the current study. However, no effect of treatment was observed. Previous work by the authors examining the EEG responses of castrated lambs in a controlled clinical setting distinguished between the responses of lambs treated with local anaesthetic and those without [13]. In the current study, there was a decrease across all EEG parameters for all treatment groups from immediately pre-procedure to immediately post-procedure. This trend has also been observed in lambs undergoing mulesing, where an overall reduction in EEG power was reported (under review, Harris et al., n.d.). The significant changes in EEG output for SC lambs across all time points may indicate a stress response to the novelty of handling and the EEG recording process. An increase in cortisol response has been reported for naïve sheep in response to handling situations including drafting, crutching and shearing [7,21]. Decreases in EEG power has been reported in response to painful procedures of mulesing and castration, and to a lesser extent from handling and shearing [17]. This was seen in the present study when examining the F50, SC values were greater than CML and C groups at all time points. The impact of lamb responses to non-painful stressors such as handling may limit the application of this method in commercial farm settings where habituation is impractical and there are uncontrollable environmental influences. However, subtle changes in F50 may be a consistent trend showing a scale of discomfort from stress of handling to painful procedure, where significance may be derived if increased artifact contamination and habituation to handling and restraint is achieved.

As with the EEG responses, environmental factors likely influenced IRT results, as a similar trend was observed over time, with no treatment interaction. Changes in eye temperature have been used to indirectly measure autonomic nervous system activation in livestock including cattle [11,22] and stress inducing scenarios in goats [23] and sheep [24,25]. Stress-induced hyperthermia occurs as an initial decrease in surface temperature followed by an increase in core body temperature as a result of a stressful stimulant [26]. This has been observed in previous work in calves as an initial decrease in eye temperature 5 min post-dehorning, followed by an increase to temperatures above baseline thereafter [11]. However, this was not the case in the present study as castrated lambs that did not receive any pain mitigation displayed an increase in eye temperature immediately after castration at 0 h. This is in line with other research in sheep, where an epinephrine infusion, with no painful intervention, resulted in an increase in eye temperature [25]. Different again are some studies in cattle undergoing castration that have found no change in eye temperature as a result of the procedure [27,28]. Different responses in eye temperature between sheep and cattle in response to painful or stressful scenarios may warrant further investigation. No changes were evident for CML lambs from baseline to 0 h. However, a significant increase in eye temp occurred at 1 and 4 h. A similar result was found in dehorned calves, where eye temperature increased 2–3 h post-procedure when local anaesthesia was expected to have worn off [12]. This increase was mitigated when calves were treated with intravenous meloxicam [12], in contrast to the current study. there was less variation in eye temperature across time for sham castrated lambs in the current study, with temperature at 24 h significantly lower than all other times including baseline. This lower 24 h temperature further indicates the stress of handling is likely indicative of a true baseline after habituation to the experimental environment has occurred.

Castrated lambs displayed significantly more abnormal behaviours overall than uncastrated lambs. Normal standing was observed in all treatment groups, though consistently occurred more for SC group over all time points. Castration has been shown to increase the time lambs spend standing and reduce the time spent lying [5,9]. There was a trend for abnormal standing to increase at 1 h, with more lambs demonstrating pain-related postures including hunching, statue standing and withdrawal from pen mates. Increases at 1 h may indicate the onset of painful inflammatory responses after the initial shock of the treatment has passed. The use of meloxicam has been seen to mitigate this response in a field study, with lambs spending less time standing than untreated lambs [9]. However, there was no treatment effect of meloxicam in the present study; behaviours of castrate lambs showed no differences regardless of pain mitigation. It is possible that in the present study, the proximity of the pens to the treatment area and the novelty of handling may have limited the expression of some behaviours such as lying and increased time spent standing for all treatment groups. Further, standing of either classification was observed more than other behaviours. Low occurrences of other behaviours could also be attributed to the impact of the experimental setting on the behaviour of lambs, with the stoic nature of sheep likely increasing vigilance and alert postures in response to fear [29].

Castrated lambs were more likely to have a score of 1 or higher for all stages of castration compared to of 0 or 1 in SC lambs. Procedural pain is difficult to address in farm settings often due to the practical necessity of a short duration between administration of analgesia and the procedure occurring. Administration of lignocaine to the spermatic cord and scrotal sac prior to castration did not abolish reactions to the procedure. Differences between castration groups were less distinguished, with CML and C groups responding similarly. Lignocaine is one of few practical options available to address procedural pain of castration in lambs. It has effectively reduced the pain of rubber ring castration in lambs, reducing behavioural responses and lowering cortisol [30]. It has also been shown to not completely remove perception in response to castration in lambs, as a study found that lignocaine reduced the pain response compared to control animals but did not eliminate it [31]. However, the efficacy of lignocaine for relief of pain caused by surgical castration in lambs seems to be more variable. A study found that lambs administered lignocaine (2 mg/kg) ten minutes prior to incision had similar increases intraoperative HR, mean arterial pressure and postoperative cortisol concentration to untreated lambs [32]. Likewise, in the current study, it did not appear that lignocaine attenuated pain responses to the procedure. This is also in line with other studies evaluating surgical castration in calves [28] and piglets [33] where pain responses were still observed in animals treated with lignocaine. Time of administration also plays a role, a longer period between treatment and castration may result in improved efficacy. However, this is impractical in a farm setting. Reduced efficacy could be a result of ineffective lignocaine administration, where injection into the spermatic cord in the correct location to impede nerve impulses and inhibit pain perception fails. Given the variation in success that this method has at reducing the pain of castration, alternative methods such as intratesticular blocks or epidural anaesthesia should be explored in this setting.

## 5. Conclusions

This study examined the use of EEG, eye temperature and behaviour to measure the response to castration in conscious lambs over a 24 h period. There were significant changes seen in EEG output, with decreasing power across treatments post-castration, though no clear effect of treatment on the outcomes measured in the current study was observed. Eye temperature increased in untreated castrated lambs immediately post-castration, with no change for CML lambs until 1 and 4 h, likely coinciding with waning analgesia. Reaction scores and behavioural observations were able to differentiate sham castrated lambs from both castration groups but a lack of variation between CML and C groups suggests insufficient analgesia to address castration pain. Pain expression can be difficult to measure in sheep and the impact of environmental stress on the outcomes measured was evident in this study. Use of EEG alone in this study did not provide sufficient evidence to detect and quantify pain. No single measure provides a complete picture of the pain response to castration, and the limitations of EEG in a farm setting may be attenuated to some degree by inclusion of multiple parameters to provide a more holistic picture of analgesic efficacy. However, the practical nature of pain assessment on farm is likely better suited to more robust measures, such as behavioural observations.

## Figures and Tables

**Table 1:** Response to castration descriptions and score outcomes.

Observation	Score	Description
Overall reaction	0	no reaction
	1	mild reaction
	2	moderate reaction
	3	strong reaction
Distal scrotal incision	0	no reaction
	1	mild reaction
	2	moderate—flinch localised to rump, back legs or slight body flinch
	3	strong—full-body flinch including front limb extension, head throws or raises
Testes	0	no reaction
	1	reaction localised to rump or lower body
	2	reaction with hind and/or front limb extension, and/or head throw/raise
	3	multiple reactions (as described above) involving whole body

**Table 2:** Ethogram used for behavioural observations for lambs after castration.

Behaviour	Description
Normal ventral lying	Lay on sternum with legs tucked in and head up or down.
Abnormal ventral lying	Ventral lying with hind limbs partially or fully extended or keeping scrotal region off the ground (dog sitting).
Ventral lying other	Lamb was lying ventrally but unable to clearly categorise the lying posture.
Lateral lying	Lateral (on side) with one shoulder on ground, extension of hind limbs with head up or down.
Normal standing	Standing with no apparent abnormalities.
Hunched standing	Lamb stood with back arched, with head lower than highest point of back.
Abnormal standing	Other abnormal standing, e.g., statue standing: immobile standing with an obvious withdrawal
	from interaction with other pen members and outside stimuli; or stretched standing: legs
	positioned further back than normal.
Normal walking	Walking with no apparent abnormalities.
Abnormal walking	Walking unsteadily or stiffly, includes walking backwards, on knees, moving forward with bunny hops, circling, leaning or falling.
Walking other	Lamb was walking but unable to clearly categorise the walking type.
Eating/drinking	Eating hay or drinking water from trough.

**Table 3:** Predicted means for the measurement parameters of median frequency (F50), 95th percentile power (F95) and total power (Ptot) with back-transformed values in parenthesis, for each treatment groups: castration + analgesia (CML), castration (C), and sham control (SC) (*n* = 8 for all treatments); for time points pre (baseline), 0, 1, 4 and 24 h post-castration. F50 did not require data transformation, and therefore model predicted means are presented. Means between time points within a treatment group (column) with differing superscripts ^ABCDE^ are significantly different.

		Treatment		
	Time	Castration + Analgesia (CML)	Castration (C)	Sham Control (SC)
F50	Pre	12.45 ^A^	12.80 ^A^	12.92 ^A^
	0 h	11.79 ^B^	12.71 ^B^	12.83 ^A^
	1 h	12.87 ^C^	12.73 ^C^	12.86 ^B^
	4 h	11.79 ^B^	12.78 ^D^	12.90 ^C^
	24 h	14.29 ^C^	12.76 ^ABD^	12.87 ^D^
F95	Pre	3.12 (22.59) ^A^	3.15 (23.28) ^A^	3.12 (22.68) ^A^
	0 h	3.09 (21.90) ^B^	3.13 (22.83) ^B^	3.13 (22.77) ^B^
	1 h	3.13 (22.88) ^C^	3.11 (22.47) ^C^	3.10 (22.31) ^C^
	4 h	3.10 (22.22) ^D^	3.14 (23.06) ^D^	3.09 (21.98) ^D^
	24 h	3.19 (24.40) ^C^	3.21 (24.66) ^A^	3.19 (21.98) ^A^
Ptot	Pre	−10.23 (3.60 × 10^−5^) ^A^	−10.19 (3.77 × 10^−5^) ^A^	−10.38 (3.10 × 10^−5^) ^A^
	0 h	−10.34 (3.22 × 10^−5^) ^B^	−10.30 (3.36 × 10^−5^) ^B^	−10.46 (2.88 × 10^−5^) ^B^
	1 h	−10.34 (3.23 × 10^−5^) ^B^	−10.57 (2.57 × 10^−5^) ^C^	−10.59 (2.51 × 10^−5^) ^C^
	4 h	−10.51 (2.73 × 10^−5^) ^C^	−10.30 (3.35 × 10^−5^) ^B^	−10.31 (3.32 × 10^−5^) ^D^
	24 h	−9.45 (7.77 × 10^−5^) ^A B^	−9.25 (9.64 × 10^−5^) ^A^	−9.11 (1.11 × 10^−4^) ^E^

**Table 4:** Probability of lambs in different treatment groups (castration + analgesia (CML), castration (C), and sham control (SC) (*n* = 8 for each treatment)) reacting to stages of castration—scrotal incision, removal of testis 1 (right), removal of testis 2 (left)—and overall reaction to castration procedure.

	Score					
Procedure	Treatment	0	1	2	3	*p* Value
Scrotal incision	CML	0.1	99.75	0.15	0	*p* < 0.0001
	C	0	0.31	99.68	0.01	
	SC	99.98	0.02	0	0	
Testis 1	CML	0	0	1	0	
	C	0	0	0	1	*p* < 0.0001
	SC	1	0	0	0	
Testis 2	CML	0	0	0	1	
	C	0	0	0	1	*p* = 0.04
	SC	1	0	0	0	
Overall reaction	CML	0	0.05	0.9	0.04	
	C	0	0.02	0.88	0.09	*p* = 0.14
	SC	0.99	0.01	0	0	

**Table 5:** Table of *p* values of model terms for behaviour data analysis using generalised linear mixed model (glmer) with binomial proportions. Fixed effects were Treatment and Time, with a random term of Lamb included. The model looked at treatment, time and their interaction. Treatment groups are as follows: castration + analgesia (CML), castration (C), and sham control (SC).

	Treatment	Time	Interaction
Normal lying	0.449	<0.001	<0.001
Abnormal lying	0.832	0.265	0.172
Normal standing	0.064	0.41	0.002
Abnormal standing	0.066	0.045	0.174
Normal walking	0.436	0.765	0.77
Abnormal walking	0.583	<0.001	0.997
Total Normal	0.079	0.553	0.076
Total Abnormal	0.017	0.373	0.553

**Table 6:** Table of back-transformed means for significant treatment x time interactions with the least significant difference presented. Treatment groups are as follows: castration + analgesia (CML), castration (C), and sham control (SC).

		Time				
Behaviour	Tx	0 h	1 h	4 h	24 h	LSD
Normal lying	CML	0.02	0.01	0.03	0.001	2.38
	C	0.03	0.004	0.02	0.02	
	SC	0.001	0.001	0.01	0.002	
Normal standing	CML	0.51	0.40	0.43	0.48	0.87
	C	0.53	0.42	0.56	0.56	
	SC	0.72	0.83	0.54	0.63	

**Table 7:** Table of back-transformed predicted means for significant effects of time, with the least significant difference presented. Treatment groups are as follows: castration + analgesia (CML), castration (C), and sham control (SC).

		Time			
Behaviour	0 h	1 h	4 h	24 h	LSD
Abnormal standing	0.20	0.29	0.21	0.24	0.44
Abnormal walking	0.003	0.0001	0.0001	0.004	1.67

**Table 8:** Table of back-transformed predicted means for significant effects of treatment, with the least significant difference presented. Treatment groups are as follows: castration + analgesia (CML), castration (C), and sham control (SC).

	Treatment			
Behaviour	CML	C	SC	LSD
Total abnormal behaviour	0.41	0.39	0.18	0.79

## Data Availability

Not applicable.
